# Mutualism promotes site selection in a large marine planktivore

**DOI:** 10.1002/ece3.7464

**Published:** 2021-03-26

**Authors:** Asia O. Armstrong, Amelia J. Armstrong, Michael B. Bennett, Anthony J. Richardson, Kathy A. Townsend, Jason D. Everett, Graeme C. Hays, Hugh Pederson, Christine L. Dudgeon

**Affiliations:** ^1^ School of Biomedical Sciences The University of Queensland St Lucia QLD Australia; ^2^ Centre for Applications in Natural Resource Mathematics (CARM) School of Mathematics and Physics The University of Queensland St Lucia QLD Australia; ^3^ CSIRO Oceans and Atmosphere Queensland Biosciences Precinct (QBP) St Lucia QLD Australia; ^4^ School of Science, Technology and Engineering University of the Sunshine Coast Hervey Bay QLD Australia; ^5^ School of Biological, Earth, and Environmental Sciences University of New South Wales Sydney NSW Australia; ^6^ School of Life and Environmental Sciences Deakin University Geelong VIC Australia; ^7^ Innovasea Hobart TAS Australia

**Keywords:** acoustic tracking, animal navigation, coral reef, elasmobranch, location accuracy, megafauna, movement ecology, VEMCO Positioning System

## Abstract

1. Mutualism is a form of symbiosis whereby both parties benefit from the relationship. An example is cleaning symbiosis, which has been observed in terrestrial and marine environments. The most recognized form of marine cleaning symbiosis is that of cleaner fishes and their clients.
2. Cleaner species set up cleaning stations on the reef, and other species seek out their services. However, it is not well understood how the presence of cleaning stations influence movements of large highly mobile species. We examined the role of cleaning stations as a driver of movement and habitat use in a mobile client species.
3. Here, we used a combination of passive acoustic telemetry and in‐water surveys to investigate cleaning station attendance by the reef manta ray *Mobula alfredi*. We employed a novel approach in the form of a fine‐scale acoustic receiver array set up around a known cleaning area and tagged 42 rays. Within the array, we mapped structural features, surveyed the distribution of cleaner wrasse, and observed the habitat use of the rays.
4. We found manta ray space use was significantly associated with blue‐streak cleaner wrasse *Labroides dimidiatus* distribution and hard coral substrate. Cleaning interactions dominated their habitat use at this site, taking precedence over other life history traits such as feeding and courtship.
5. This study has demonstrated that cleaning symbiosis is a driver for highly mobile, and otherwise pelagic, species to visit inshore reef environments. We suggest that targeted and long‐term use of specific cleaning stations reflects manta rays having a long‐term memory and cognitive map of some shallow reef environments where quality cleaning is provided. We hypothesize that animals prefer cleaning sites in proximity to productive foraging regions.

## Graphical abstract

This study investigates the role of mutualism in determining site selection in marine megafauna. The aim was to determine the role of cleaning stations as a driver of movement and habitat use in a mobile client species. Through a combination of in‐water observations and a novel application of fine‐scale passive acoustic tracking, we demonstrated that cleaning symbiosis is a driver for highly mobile, and otherwise pelagic species to visit inshore reef environments. We suggest that targeted and long‐term use of specific cleaning stations reflects manta rays having a long‐term memory and cognitive map of some shallow reef environments where quality cleaning is provided. We hypothesize that animals preference cleaning sites in proximity to productive foraging regions.

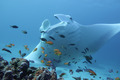

## 1 INTRODUCTION

Mutualism is the exchange of goods and services between organisms that provides a net benefit to those involved. A classic example is pollination, in which an animal vector receives food in the form of pollen or nectar, in exchange for fertilizing a plant's ovules (Cushman & Beattie, 1991). Another is cleaning symbiosis, whereby a client species has ectoparasites removed by a host cleaner species (Limbaugh, 1961). On land, this symbiosis is commonly observed in bird species such as oxpeckers *Buphagus* spp. removing ticks and blood‐sucking flies from ungulates (Sazima, 2011). Cleaning symbiosis is also found, though less commonly, in other taxa including small mammals: an example is the banded mongoose *Mungos mungo* removing ticks from common warthogs *Phacochoerus africanus* (Sazima, 2010). In the ocean, over 100 marine fishes and numerous invertebrate species act as cleaners to a wide range of taxa including cephalopod mollusks, fishes, mammals, and reptiles (Côté, 2000). Host cleaner species in marine systems are generally more site attached than their terrestrial counterparts, setting up “cleaning stations” that clients seek out and visit. However, to date there is little understanding of how this mutualism promotes site selection in large‐bodied, vagile marine species.

Many species of marine megafauna have extensive home ranges, moving 100s of kilometers in search of food or undergoing reproductive migrations (e.g., leatherback turtles *Dermochelys coriacea*, sperm whales *Physeter macrocephalus*, and lemon sharks *Negaprion brevirostris*; (Christal & Whitehead, 1997, Houghton et al., 2006, Chapman et al., 2009). Yet large migratory species also seek out the services of site‐attached cleaners (e.g., oceanic sunfish *Mola mola*, pelagic thresher sharks *Alopias pelagicus*, whale sharks *Rhincodon typus*, and manta rays *Mobula birostris* and *M. alfredi*; (Konow et al., 2006, O’Shea et al., 2010, Oliver et al., 2011, Araujo et al., 2020, Murie et al., 2020). Cleaning interactions typically involve the cleaner removing ectoparasites from visiting or resident clients, but cleaners may also feed on host mucus and skin, particularly at wound sites (Grutter, 1999). The ecological importance of this symbiosis in terms of body maintenance, especially for mobile client species, has been demonstrated on tropical coral reefs via experimental exclusion of the blue‐streak cleaner wrasse, *Labroides dimidiatus*. For client species, this has resulted in decreased diversity and abundance, higher rates of fungal infection, smaller body size, and poorer health (Bshary, 2003; Bshary et al., 2007; Grutter et al., 2003; Waldie et al., 2011). Mobile client species may therefore benefit from their choice to visit reefs based on the presence of cleaner fish. In turn, reef‐associated cleaners benefit from large, mobile species bringing a food source that originates from off the reef, suggesting that this symbiosis is indeed mutualistic.

The preference of mobile species for particular sites indicates they have a spatial memory of the sites and the service they have received. The species of cleaner fish at a site may influence the quality of the cleaning station, and large mobile marine species have multiple options when it comes to habitat choice. Mobile clients would likely opt for cleaning stations where they receive quality service (i.e., parasite removal with few or no adverse events, such as biting from the cleaners; (Bshary & Schäffer, 2002)) and would be less likely to return to a site where they were previously not attended to promptly (Bshary & Grutter, 2002). Perhaps then it is not surprising that clients with multiple choices of cleaning stations are given better service than clients with reduced options, suggesting that cleaners can distinguish between resident and visiting clients (Adam, 2010). A key determinant of a mobile client's attendance at a particular cleaning station site may be the memory of quality cleaning service and the animal's spatial cognition. How animals move in relation to learning and memory remains a key question in megafauna studies (Hays et al., 2016), and the role of spatial cognition in relation to fine‐scale habitat use is not well understood.

Many large, mobile elasmobranch species have been observed attending cleaning stations, including spotted eagle rays *Aetobatus narinari*, pelagic thresher sharks, silky sharks *Carcharhinus falciformis*, Galapagos sharks *C. galapagensis*, bull sharks *C. leucas*, demersal lemon sharks *Negaprion acutidens*, whale sharks, and scalloped hammerheads *Sphyrna lewini* (Keyes, 1982; Oliver et al., 2011; Quimbayo et al., 2016). Ectoparasite loads may drive these visits, as individuals with high parasite loads frequent cleaning stations more regularly than individuals with low parasite loads (Grutter, 1999). Ectoparasite loads are lower after cleaning interactions (Keyes, 1982), and cleaner fish spend longer foraging on body regions with more ectoparasites (Oliver et al., 2011). Larger elasmobranchs may also receive preferential treatment from the cleaner community (Keyes, 1982; Oliver et al., 2011). Larger fish with more parasites are inspected more often and for longer than smaller fish with fewer parasites (Grutter, 1995). Further, facultative cleaners favor interactions with planktivore clients over piscivores, which is likely to be related to a lower risk of being eaten by the client fish (Francini‐Filho & Sazima, 2008). However, drivers of site selection by large mobile clients at cleaning station habitats have not previously been investigated.

**FIGURE 1:**
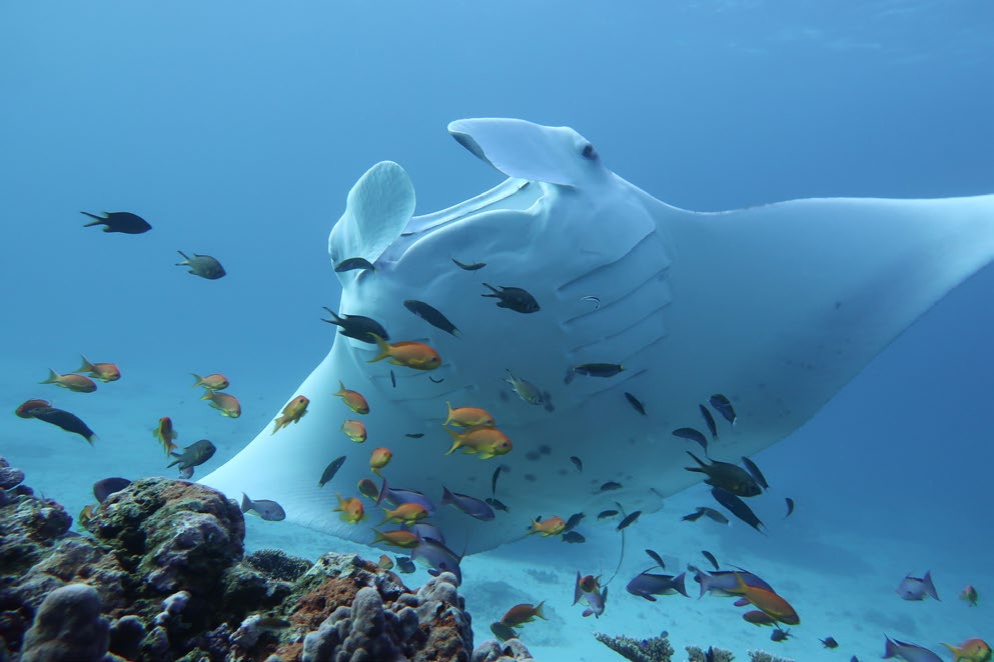
Reef manta ray *Mobula alfredi* attending a cleaning station at Lady Elliot Island, Australia

The reef manta ray is a large, mobile planktivorous species that demonstrates site affinity to reef environments, including cleaning stations (Figure 1). This affinity has been documented at aggregation sites in the Maldives (Stevens, 2016), eastern Australia (Couturier et al., 2018), Indonesia (Germanov et al., 2019), the Seychelles (Peel, Stevens, et al., 2019), and Mozambique (Venables et al., 2020). These studies have documented that manta rays visit cleaning stations, but the methods used in most studies have limitations for determining behavior and fine‐scale spatial use. For example, while observations by SCUBA or free divers or via remote underwater cameras can provide fine‐scale information, they are limited to short observational windows. By contrast, aquatic telemetry approaches such as acoustic or satellite tagging can facilitate continuous, long‐term detections, but typically lack spatial resolution. However, combining these approaches, and enhancing the fine‐scale tracking of animal movements, could elucidate the site preferences and drivers of mobile client's visitation to particular sites.

Here, we investigated the role of mutualism in determining site selection in vagile marine megafauna. Our aim was to determine the role of cleaning stations as a driver of movement and habitat use in a mobile client species. Through a combination of in‐water observations and a novel application of fine‐scale passive acoustic tracking, we show how high‐accuracy tracking can elucidate the memory of particular shallow reef habitats and space use in a largely pelagic species and provide a mechanism for nutrient exchange between reef and open ocean environments.

## 2 MATERIALS AND METHODS

### 2.1 Study site

**FIGURE 2:**
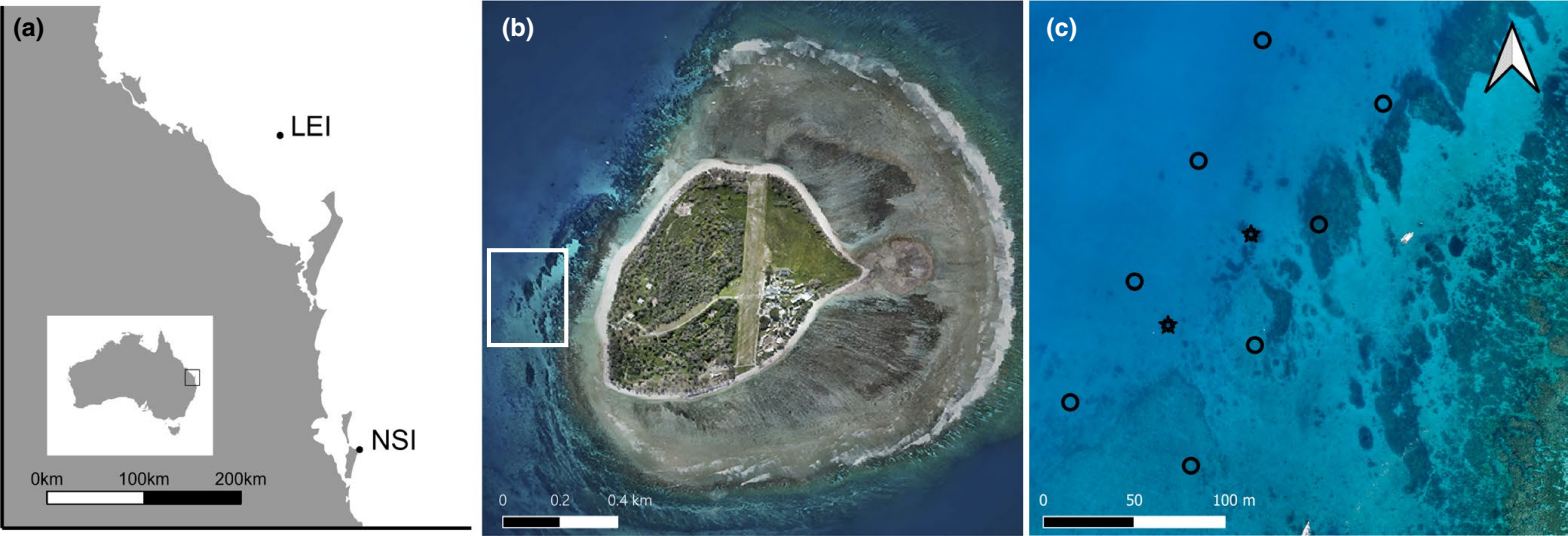
Key *Mobula alfredi* sites in eastern Australia. (a) Two aggregation sites of *M. alfredi* in southern Queensland, LEI = Lady Elliot Island and NSI = North Stradbroke Island; (b) Aerial view of LEI, white boxed area highlighting the study region; and (c) Zoomed in view of the study region on the western side of LEI. Black circles indicate positions of the acoustic receivers, and black stars indicate the location of temperature loggers and sentinel transmitters (Image credit: Jeremy Somerville)

*Mobula alfredi* is found in tropical and subtropical waters of the Indo‐Pacific and Indian Ocean. In Australia, it is found in coastal waters north of ~30°S (Figure 2a; Armstrong et al., 2020), with the largest known aggregation on the east coast around Lady Elliot Island (LEI, Figure 2b; Couturier et al., 2014). The peak of the *M. alfredi* aggregation at LEI is in winter, and during summer many individuals migrate south, with North Stradbroke Island (Figure 2a) a seasonal aggregation site (Couturier et al., 2011). At LEI, previous research identified a high‐use area on the western side of the island where *M. alfredi* individuals cruise, feed, court, and are cleaned (Couturier et al., 2018; Jaine et al., 2012). This area has a series of coral reef features 8–15 m deep (Figure 2c).

### 2.2 Acoustic tracking

To monitor the presence and location of acoustically tagged *M. alfredi* on the western side of LEI, an array of eight acoustic receivers (VR2W; Vemco, Nova Scotia) were deployed as a VEMCO Positioning System (VPS) between February 2017 and October 2018 (Figure 2c). Receivers were ~76 m apart in a grid formation placed at GPS‐verified locations. Each receiver was paired with an acoustic transmitter or sync tag, attached 1 m above the VR2W, which emitted a unique coded pulse‐train every 500–700 s. These transmissions allowed for the relative position of each receiver within the array to be determined throughout the study, to provide an estimate of the location error associated with the calculated positions of tagged animals. To assess the magnitude of the positioning error, two sentinel transmitters and temperature loggers were placed at GPS‐verified locations within the array for the duration of the study. The VPS facilitates precise locational tracking of tagged animals in and around the acoustic array (Espinoza et al., 2011; Roy et al., 2014), and receivers in the LEI array were set up conservatively (close together) to ensure stable performance over a 24‐hr cycle.

A total of 42 individual *M. alfredi* (27 female, 15 male) were tagged with V16 acoustic transmitters (Vemco, Nova Scotia) with a random delay of 60–90 s (Table A1). Each transmitter was attached to a Domeier umbrella‐dart tag head with a 10 cm shrink‐wrapped braided wire tether and was inserted into the dorsal musculature of a pectoral fin of a free‐swimming ray using a modified Hawaiian sling spear. Antifoul was not applied due to the relatively short retention times of external Domeier umbrella‐dart head tags on manta rays (median detection time of 121 days; Couturier et al. 2018). Prior to tagging, individual *M. alfredi* were photographed for subsequent identification by comparing the image with those in an existing photo‐ID database (Armstrong et al., 2019). Sex was assessed based on the presence (male) or absence (female) of claspers (Marshall et al., 2011). Maturity status was determined by the clasper size (in males), and pregnancy and/or presence of mating scars (in females; Marshall & Bennett, 2010). Animal size was estimated visually to the nearest 0.5 m using stationary objects for scale, and all females were assigned as adult if their disk width was ≥3.5 m (Couturier et al., 2014). Tagging commenced in February 2017 at North Stradbroke Island (*n* = 10), and the remaining tags were deployed at LEI between February 2017 and June 2018 (*n* = 32).

### 2.3 Processing acoustic data

The position of a tagged *M. alfredi* within the array was calculated from the time‐difference‐of‐arrival of its transmitter pulse‐train at each receiver (minimum three receivers required to calculate a position estimate). The process relies on knowing the precise relative positions of the receivers and their sync tags. For all position estimates (animal transmitters and sync tags), a relative Horizontal Positioning Error was calculated based on ranges of water temperature, depth, and salinity of the particular VPS, as well as the geometry of the transmitter and detecting receivers for specific transmissions (VEMCO, 2018). For each sync tag position estimate, a measured Horizontal Positioning Error value (in meters) is also calculated, as their true location is known. Using the relationship between the Horizontal Positioning Error values and the measured Horizontal Positioning Error values from the sync tags, we extrapolated measured Horizontal Positioning Error values for animal transmitters (Coates et al., 2013). We could then provide an estimate (in meters) of the error associated with each animal's transmitter position. To improve precision of position estimates and provide confidence in our conclusions regarding manta ray space use at the site, we removed 10% of the positions with the greatest measured Horizontal Positioning Error (Roy et al., 2014). Sentinel transmitter positions were used as a control to reduce the potential for making erroneous inferences regarding animal behavior (Payne et al., 2010) and to rule out the possibility that animal position estimates were heavily influenced by background reef noise.

### 2.4 In‐water observations

Stationary point surveys of *M. alfredi* cleaning interactions with host cleaner fish were made between June 2017 and June 2019 (*n* = 67). Surveys were conducted for 10 min by two SCUBA divers at four locations within the acoustic array cleaning area, during which the abundance and diversity of cleaner fish species that interacted with *M. alfredi* was recorded. Each dive (~60 min) could have up to four surveys, allowing for transit time between locations. There were regularly two dives in one day (one in the morning, one in the afternoon). The maximum number of cleaner fish counted per survey (MaxN) was used in subsequent analyses, as this alleviates concerns that an individual may be counted more than once if it leaves the observers field of view (Bosch et al., 2017). Locations were chosen based on previous observations of manta rays cleaning at the site. A manta ray was deemed to be getting cleaned when two criteria were met: (a) it approached the cleaning station (to within ~2 m) and maintained position or started circling the station for more than one minute; and (b) it had cleaner fish attending it. If *M. alfredi* was encountered within the array, but not at a cleaning station, an image was taken for photo‐ID and behavior recorded as feeding, cruising (as defined by Jaine et al., (2012)), or courtship (as defined by Stevens (2016)). Photo‐ID was used to ensure individuals were only documented once during each SCUBA dive (~60 min) and that their dominant behavior was recorded.

**FIGURE 3:**
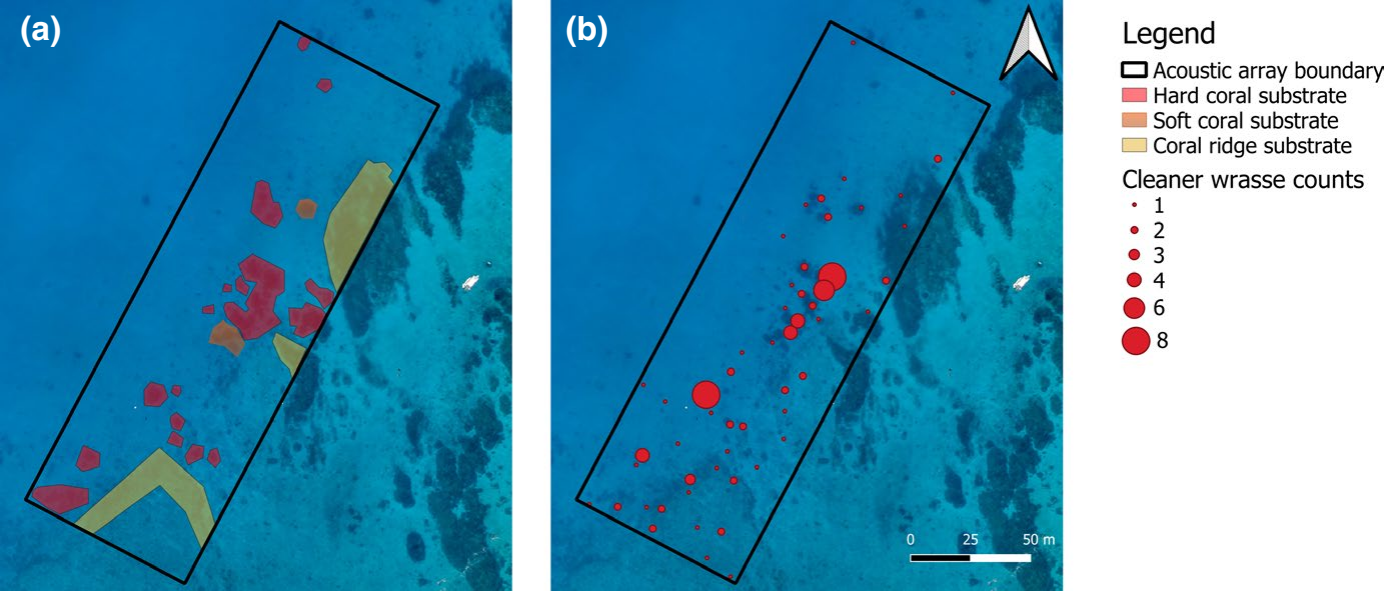
Distribution of structural features and cleaner wrasse within an acoustic array off Lady Elliot Island. (a) Colored polygons represent different substrate types, with the remaining regions being sand or structural features <2 m in diameter; and (b) Distribution and abundance of blue‐streak cleaner wrasse, *Labroides dimidiatus* (Image credit: Jeremy Somerville)

**FIGURE A1:**
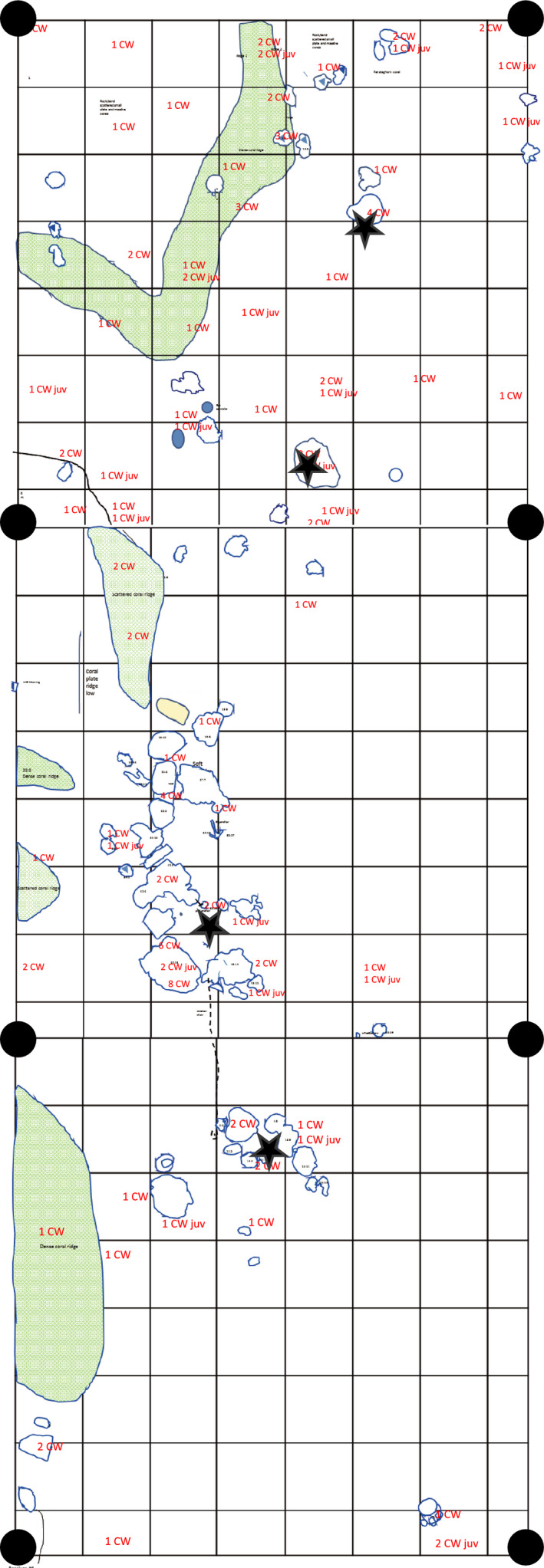
Mapped structural habitat within the Lady Elliot Island acoustic array. The mapped area was divided into three large squares (76 × 76 m) using the position of the acoustic receivers (seen here as black circles). Mapping was conducted using transect tape within 10 × 10 m gridded squares (size adjusted along the bounds of the larger 76 × 76 m square), and photographs of features were taken to confirm structural habitat. Features <2 m were excluded from mapping. Stationary point surveys for cleaner species were conducted at locations marked with a black star

To investigate associations among substrate type, cleaner fish and *M. alfredi*, we mapped the habitat and the obligate cleaner *L. dimidiatus* within the boundary of the acoustic array (Figure 3). For the habitat mapping, two divers on SCUBA conducted gridded surveys using transect tapes and photographs to create a map of reef features (Figure A1). Transect tapes were laid out to form 10 m by 10 m grid squares using a compass to maintain direction, and all features >2 m in diameter were measured and plotted on the gridded map. Substrate types were categorized as sand (bare sand and <2 m diameter sparsely distributed corals), hard coral, soft coral, or dense coral ridge. To map the distribution of cleaner fish within the acoustic array, we conducted three surveys on SCUBA using the aforementioned habitat map to provide accurate positions for spatial analysis. Counts *of L. dimidiatus* were obtained and cross‐checked by two divers on SCUBA. Surveys were conducted in June to coincide with the observed peak of *M. alfredi* visits to the study site (Couturier et al., 2011). The focus of the distribution surveys was the obligate cleaner fish, *L. dimidiatus*, as this species is found across all habitat zones (Green, 1996). *Labroides dimidiatus* establishes cleaning stations at fixed locations (Potts, 1973), whereas the other cleaner species observed in the current study are facultative cleaners, and less site attached.

### 2.5 Statistical analyses

**FIGURE A2:**
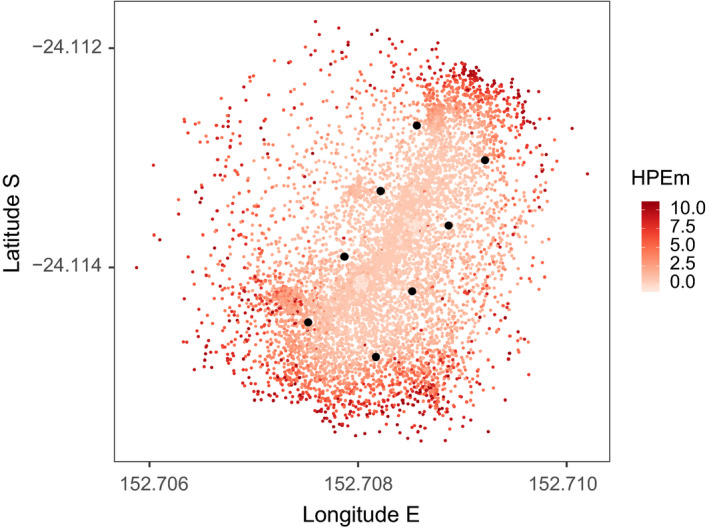
Error estimate of tagged *Mobula alfredi* positions in an acoustic array. The colored dots represent 90% of tagged animal positions (*n* = 12,157) after removing positions with lower location accuracy (measured Horizontal Positioning Error (HPEm) over 10.82 m). Black circles indicate location of acoustic receivers

To quantify the fine‐scale habitat use of *M. alfredi*, we excluded position estimates calculated outside the acoustic array boundary and focused on the location of tagged animals within the array. The region within the array boundary is where positional accuracy of tagged *M. alfredi* was highest (Figure A2), and where we mapped structural features and *L. dimidiatus* locations. Positions of individual *M. alfredi*, *L. dimidiatus*, and substrate polygons were transformed into spatial data using a 5 × 5 m grid in the R packages “raster” (Hijmans, 2020) and “sf” (Pebesma, 2018). To explore associations between locations of *M. alfredi* and substrate types, and between locations of *M. alfredi* and *L. dimidiatus*, we used a modified *t* test in the R package “SpatialPack” (Osorio & Vallejos, 2019).

Cleaning area visitation events for tagged *M. alfredi* were calculated using the R package GLATOS (Holbrook et al., 2017). A visitation event was based on a minimum of two position estimates in and around the acoustic array to allow for animals in transit. Visitation events were deemed separate if the time between position estimates exceeded 300 s.

To investigate potential drivers of *M. alfredi* visits around the VPS array, we constructed models in the R package “lme4” (Bates et al., 2014). To test for significant predictors, we used the R package “lmerTest” (Kuznetsova et al., 2017). We conducted analyses using two different response variables: (a) Count (the number of *M. alfredi* position estimates per hour); and (b) Duration (the length of each *M. alfredi* visitation event in s). For the Count model, we used a generalized linear model (GLM) with a negative binomial error structure (as a Poisson error structure was overdispersed), and for the Duration models we used a generalized linear mixed effect model (GLMM) incorporating individual tagged *M. alfredi* as a random effect with a Gamma error structure (as the variance generally increased with the square of the mean and there were zeros present). Both models had a log‐link function and were constructed using the glm.nb and glmer function for the Count and Duration models, respectively, in the R package “lme4” (Bates et al., 2014). During model development, we visually inspected diagnostic plots to assess assumptions of homogeneity of variance and normality.

**FIGURE A3:**
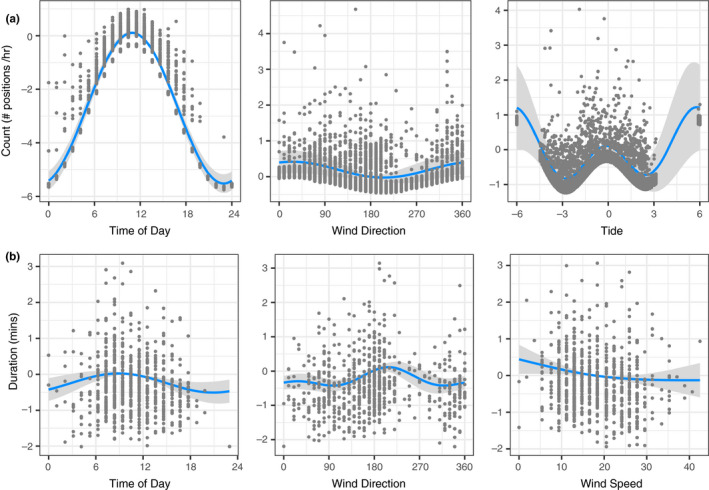
Generalized linear mixed effects models of tagged *Mobula alfredi* at Lady Elliot Island cleaning stations: (a) Count per hour of detections by Time of Day (0–24 hr), Wind Direction (South = 180°, North = 360°), and Tide (Hours from low tide at 0); and b) Duration in minutes of visitation by Wind Speed (km/hr), Wind Direction (South = 180°, North = 360°), and Tide (Hours from low tide at 0). Output is on the log‐link scale

Predictors in the Count model were Time of Day (continuous), Tide (continuous), Wind Direction (continuous), and Wind Speed (continuous). Fixed effect predictors in the Duration model were Time of Day (continuous), Sex of the Ray (factor), Maturity (factor), Size Estimate (continuous), Tide (continuous), Wind Direction (continuous), and Wind Speed (continuous). Individual ray attributes were not appropriate for the Count model as this dataset was summarized by hour and there were zeros present. Environmental information was obtained from the Australian Bureau of Meteorology. Tide was calculated as the hours from low tide. To account for the circular nature of Tide (~12‐hr cycle), Wind Direction (360° cycle), and Time of Day (24‐hr cycle), variables were transformed using a truncated Fourier series (a harmonic function of sine and cosines). This ensures that the cyclical nature of these predictors is captured, while guaranteeing that the response values predicted at the extremes of the predictor range are the same (e.g., the same prediction for Count at times of 0 and 24 hr). Wind Speed was smoothed using a natural spline in the R package “splines” (R Core Team, 2019). Explained deviance was calculated in R using delta values in the package “MuMIn” (Barton, 2009). Final models were selected based on AIC values, and significance of variables was taken at *p* < .05. To visualize the generalized linear mixed effects models, we present contrast plots using the response scale in the R package “visreg” (Breheny & Burchett, 2017). Model output with confidence limits on the response scale is provided in the Results, and output on the log‐link scale with residuals is available in the appendix (Figure A3).

## 3 RESULTS

### 3.1 Acoustic tracking data

**FIGURE A4:**
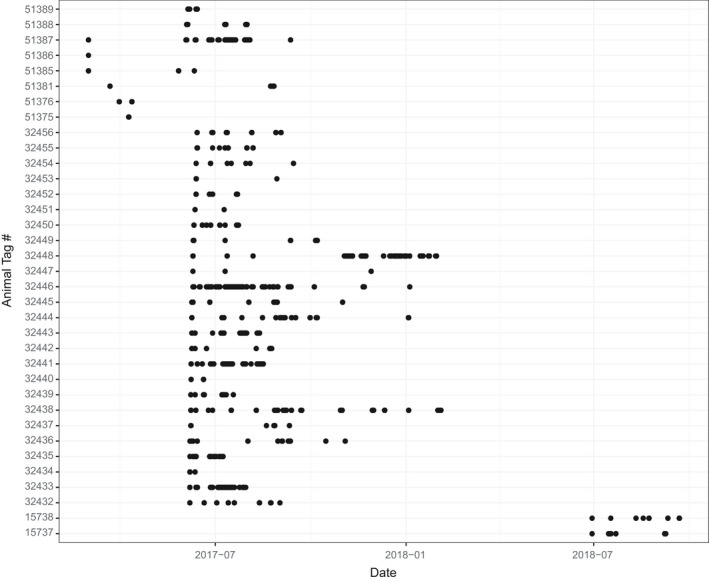
Abacus plot of tagged *Mobula alfredi* detections in an acoustic array off Lady Elliot Island. Each row represents a tagged animals detections over time

Between February 2017 and September 2018, 34 of the 42 tagged *M. alfredi* were detected multiple times by the VPS on the western side of LEI, with 114,575 detections in total. From these detections, 13,507 unique *M. alfredi* position estimates were calculated. It is the position estimates that subsequent analyses are based on. We removed 10% of the positions with the greatest measured Horizontal Positioning Error (>10.82 m), leaving 12,157 positions for subsequent analysis (Females *n* = 7,465, Males *n* = 4,692). Error estimates for the remaining animal positions were relatively small (<1.1 m for 50% of animal position estimates and <2.9 m for 75% of animal position estimates; Figure A2). There was a mean of 357.5 positions per tagged *M. alfredi* (*SD* = 344.8, Range = 29–1603), and tagged animals were detected over a mean duration of 92.3 days (*SD* = 68.4 days, Range = 5–241 days). The greatest number of consecutive days an individual was detected by the acoustic array was 11 days (Figure A4).

**FIGURE 4:**
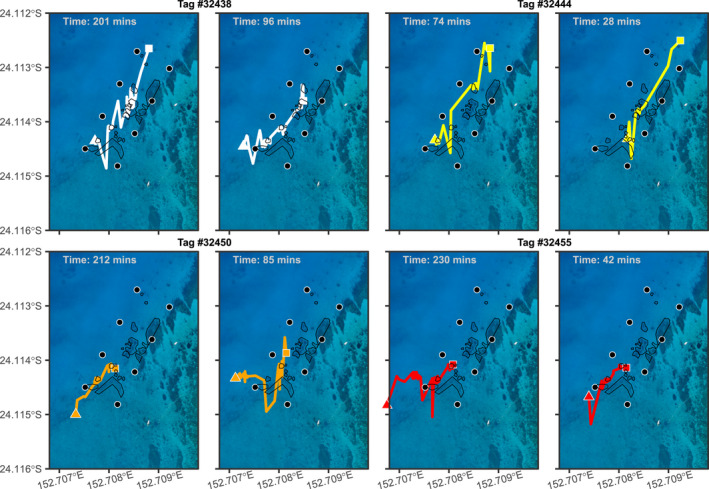
Eight examples of fine‐scale tracking of *Mobula alfredi* in the acoustic array off Lady Elliot Island. Each color represents an individual manta ray, and each plot a separate visitation event. The colored triangle denotes the start of the track, and the square is where the track ends. Black circles represent the locations of the acoustic receivers, and the black outlines are the mapped reef structures

There were 741 *M. alfredi* visitation events during the study, with a mean duration of 19.9 min (*SD* = 24.1 min, Range = 1.5–230.0 min). The mean duration for female visitation events was 21.0 min (*n* = 432), and 18.3 min for male visitation events (*n* = 309). For all animals, the median visitation duration was 12.8 min, as the data were heavily right‐skewed. There was variation in the space use within the array during visitation events, both for the same individual and among individuals, here illustrated by eight visitation events (Figure 4). Here, we observe some individuals moved less decisively between different reef features in the array (e.g., Tag #32438; Figure 4), whereas other individuals moved more directly, with their time in the array focused at particular sites (e.g., Tag #32455; Figure 4).

**FIGURE 5:**
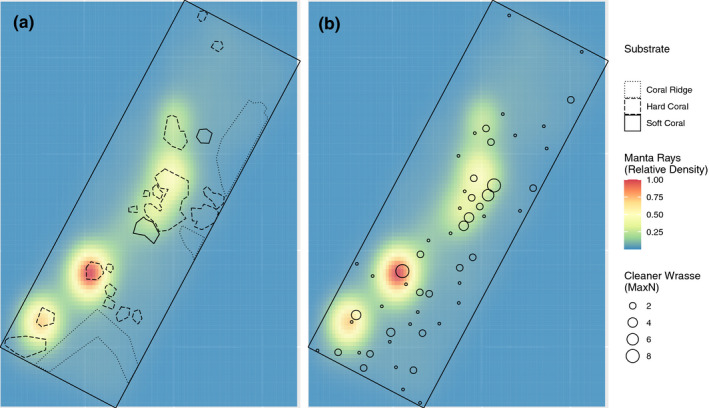
Habitat use of tagged *Mobula alfredi* in the Lady Elliot Island acoustic array between February 2017 and September 2018. (a) Relative density of manta ray positions overlaid with structural features of the substrate; and (b) Relative density of manta ray positions overlaid with density of cleaner wrasse denoted by size of black circles. The black rectangle in the figures represents the bounds of the acoustic array

**FIGURE A5:**
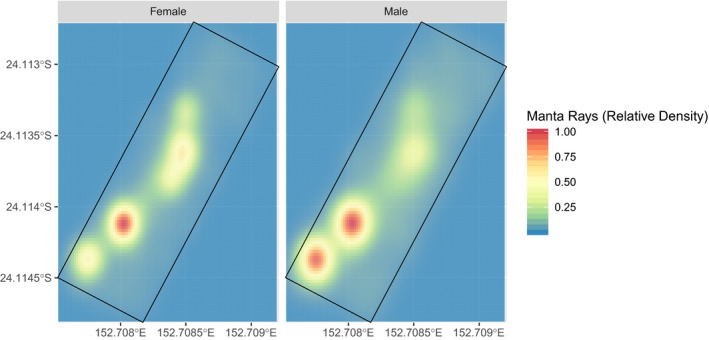
Habitat use of tagged *Mobula alfredi* in the Lady Elliot Island acoustic array between February 2017 and September 2018. Relative density of manta ray positions, with panels separated by sex of the tagged rays. The black rectangle in the figures represents the bounds of the acoustic array

A total of 6,973 positions fell within the boundary of the VPS array (57.4%). The relative density of these tagged *M. alfredi* positions was highest around structural features (Figure 5a), and where *L. dimidiatus* numbers were highest (Figure 5b). *Mobula alfredi* position was significantly associated with structural features within the array (modified *t* test, *F* = 6.03, *df* = 1, 247, *p* = .015, *r* = .15, Figure 5a). Post hoc analysis of substrate types found *M. alfredi* position was significantly associated with hard coral structure >2 m in diameter (modified *t* test, *F* = 27.47, *df* = 1, 263, *p* < .001, *r* = .31), but not with soft coral structure or the coral ridge structures. In terms of the association with cleaner fish, *M. alfredi* position was significantly related to the position of *L. dimidiatus* (modified *t* test, *F* = 49.15, *df* = 1, 510, *p* < .001, *r* = .30, Figure 5b). Overall, *L. dimidiatus* position was significantly associated with hard coral features >2 m (modified *t* test, *F* = 48.97, *df* = 1, 488, *p* < .001, *r* = .30), but not with soft coral features or coral ridge features. There was some variation in the space use between male and female *M. alfredi* (Figure A5); however, there was no difference in their association with structural features or *L. dimidiatus*.

### 3.2 In‐water observations

Surveys (*n* = 67) confirmed that the cleaner fish community for *M. alfredi* at the four identified cleaning stations was dominated by the obligate and site‐attached cleaner *L. dimidiatus* (mean = 3.6, *SD *± = 3.1), and the facultative, less site‐attached cleaner, the moon wrasse *Thalassoma lunare* (mean = 11.9, *SD*± = 7.2), with occasional inspections by the bicolor wrasse *L. bicolor* (mean = 0.2, *SD*± = 0.6), brown butterflyfish *Chaetodon kleinii* (mean = 0.4, *SD*± = 1.4), and birdnose wrasse *Gomphosus varius* (mean = 0.1, *SD*± = 0.3). The four identified cleaning stations comprised hard coral substrate and were the larger, more prominent reef features (5 + m wide and 2 + m high) in the array. There were 99 M*. alfredi* encounters during in‐water photo‐ID surveys, comprising 79 identified individuals. The majority were cleaning (81%, *n* = 80), with relatively few feeding (6%, *n* = 6), cruising (3%, *n* = 3) or courting (10%, *n* = 10).

### 3.3 Drivers of Mobula alfredi visitation

**FIGURE 6:**
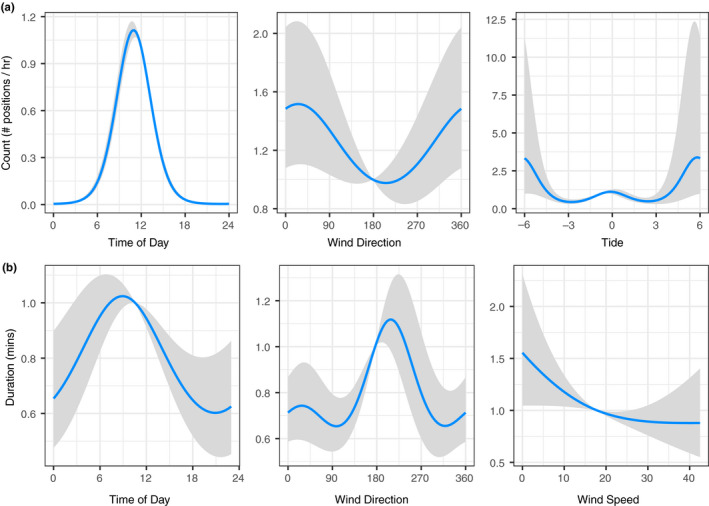
Models of tagged *Mobula alfredi* at Lady Elliot Island cleaning stations: a) Count per hour of positions by Time of Day (0–24 hr), Wind Direction (South = 180°, North = 360°), and Tide (Hours from low tide at 0); and b) Duration in minutes of visitation by Wind Speed (km/hr), Wind Direction (South = 180°, North = 360°), and Tide (Hours from low tide at 0). Output on the y‐axis is the response scale

The Count of *M. alfredi* positions from the VPS array was significantly related to Time of Day (*p* < .001), Wind Direction (*p* = .01), and Tide (*p* < .001; Figure 6a), while Wind Speed was not significant (*R* ^2^ = 0.26; Table 1). The Count of *M. alfredi* positions was highest during daylight hours, when winds were northerly, and around high and low tides.

**TABLE 1:** Model selection table for Count of *Mobula alfredi* positions (C1‐C4) and Duration of *M. alfredi* visitation events (D1‐D6). The *df* is the degrees of freedom of the Fixed effects in the model. Deviance (%) is the Total Explained Deviance from the model, with the bracketed value the Explained Deviance from the Fixed effects in the model. AIC = Akaike information criterion. Variables were removed in a stepwise approach based on AIC. The original model and the top 5 models based on AIC are displayed. The final model of each set is highlighted in bold

Model	Variables	*df*	*R* ^2^	Deviance (%)	AIC
C1	TimeofDay(k = 1)+WindDirection(k = 1)+Tide(k = 2)+WindSpeed(*df* = 2)	10	0.26	—	13,233
C2	WindDirection(k = 1)+Tide(k = 2)+WindSpeed(*df* = 2)	8	0.26	—	13,852
C3	TimeofDay(k = 1)+WindDirection(k = 1)+WindSpeed(*df* = 2)	6	0.25	—	13,249
**C4**	**TimeofDay(k = 1)+WindDirection(k = 1)+Tide(k = 2)**	**8**	**0.26**	**—**	**13,233**
D1	Sex + Maturity+SizeEstimate + TimeofDay(k = 2)+WindDirection(k = 2)+Tide(k = 2)+WindSpeed(*df* = 2)	17	—	15.6 (7.1)	5,641.5
D2	Sex + Maturity+TimeofDay(k = 2)+WindDirection(k = 2)+Tide(k = 2)+WindSpeed(*df* = 2)	16	—	15.6 (7.1)	5,639.5
D3	Sex + Maturity+TimeofDay(k = 2)+WindDirection(k = 2)+WindSpeed(*df* = 2)	12	—	14.5 (6.2)	5,639.8
D4	Sex + TimeofDay(k = 2)+WindDirection(k = 2)+WindSpeed(*df* = 2)	11	—	14.4 (5.9)	5,638.5
D5	Sex + TimeofDay(k = 1)+WindDirection(k = 2)+WindSpeed(*df* = 2)	9	—	14.3 (5.8)	5,636.2
**D6**	**TimeofDay(k = 1)+WindDirection(k = 2)+WindSpeed(*df* = 2)**	**8**	**—**	**14.2 (5.1)**	**5,635.4**

The Duration of *M. alfredi* visitation to the VPS array was significantly related to Time of Day (*p* < .001), Wind Direction (*p* < .001), and Wind Speed (*p* = .01; Figure 6b). The Duration of *M. alfredi* visitation was not related to Tide, Size Estimate, Maturity, or Sex of the Ray (Table 1). Manta rays visited cleaning stations for longer periods during daylight hours, when winds were from a SSW direction and the wind speed was low (Total explained deviance = 14.2%, Fixed effects = 5.1%, and Random effects = 8.9%).

**FIGURE A6:**
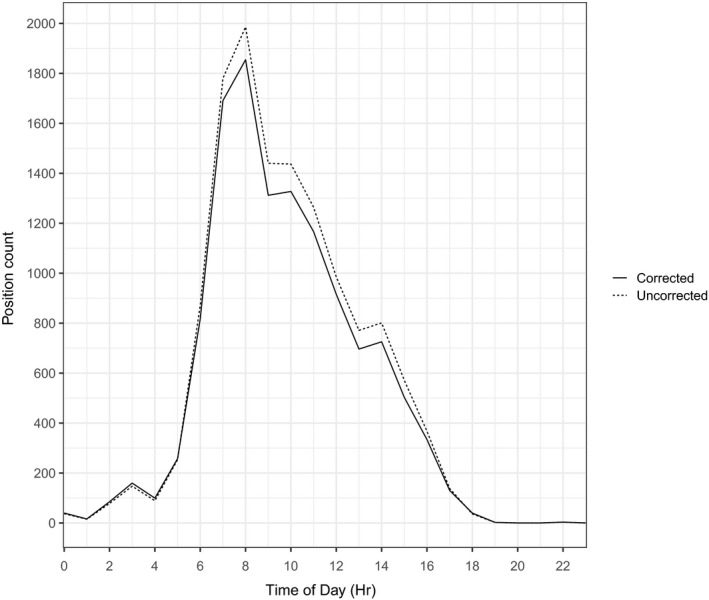
Corrected counts for tagged animal positions in an acoustic array off LEI. Using the standardized positioning frequency of sentinel transmitters, the solid line provides a corrected count per hour of animal positions from the acoustic array. The dashed line is the uncorrected count of animal positions from the array

Analysis of sentinel transmitter positions revealed that the minimal performance of the array decreased slightly at night. The correction to the total hourly counts of animal positions using the standardized positioning frequency of the sentinel transmitters did not alter the overall pattern of attendance to the site from tagged manta rays (Figure A6). We can thus be confident that the patterns observed are driven by the physics and biology, rather than an artifact of under‐performance in the acoustic array due to reef noise.

## 4 DISCUSSION

We found that mutualism—in the form of cleaning symbiosis—can promote site selection in a large‐bodied, mobile marine species. Fine‐scale site selection by a client species was associated with the distribution of the obligate cleaner *Labroides dimidiatus* and the hard coral structures where this site‐attached species has established cleaning stations. We found that interactions with cleaner species were the most commonly observed behavior in the client, taking priority over other traits such as foraging and reproductive behavior at these sites.

Generally, movement studies focus on how a few key issues, including foraging, predator avoidance, and breeding, influence animal movements (Hays et al., 2016). Set against this backdrop, our results show how an important function of daily movements may also be body maintenance, with manta rays regularly visiting cleaning stations. This regular use of cleaning stations is common in many vagile marine species, that includes bony fishes, sea turtles and elasmobranchs (Araujo et al., 2020; Konow et al., 2006; Murie et al., 2020; Oliver et al., 2011; Schofield et al., 2017). The relationship between pelagic species and their cleaners could be a common form of mutualism that controls the fine‐scale habitat use of many species and deserves more consideration. Similar to cleaning excursions, other marine taxa may take a break from foraging for other forms of body maintenance. For example, some fish that forage below the thermocline come to the surface to rewarm (Evans et al., 2014; Pope et al., 2010), whereas pinnipeds haul‐out ashore to rest (Andrews‐Goff et al., 2010; Hamilton et al., 2018). While the amount of time spent in the vicinity of cleaning stations may be small in absolute terms (tens of minutes over a 24‐hr period), it appears to be a key component of the daily time‐budget for manta rays. Similarly, many terrestrial species spend time each day grooming to remove parasites (e.g., chimpanzees (Foster et al., 2009; Lehmann & Boesch, 2008), with an important distinction being that in these cases animals do not need to travel to specific locations to undertake such behavior.

Quality cleaning habitat is an important driver of pelagic species visitation to inshore reefs. Here we established that the number of individual *L. dimidiatus* present at a particular cleaning station may influence manta ray site preference, demonstrating the disproportionate effect that a small and sparsely distributed species can have on coral reef communities (Waldie et al., 2011). *Labroides dimidiatus* is ubiquitous in tropical reef systems globally (Green, 1996), however our findings suggest that its importance in attracting pelagic visitors to the reef may have been previously underrepresented. A remaining question in the movement ecology of marine megafauna is; can movement data provide information on the ecosystem role of marine megafauna (Hays et al., 2016)? Information provided by movement studies on the spatiotemporal patterns of abundance and behaviors (e.g., foraging and cruising) of animals is key to understanding their ecological roles. For the reef environment, it may be that visits to cleaning stations from animals that spend considerable time in the open ocean (e.g., pelagic thresher sharks (Oliver et al., 2011) and manta rays (Murie et al., 2020)), provides a mechanism for nutrient exchange between these environments. This is similar to the broader‐scale example of large whales (baleen and sperm whales) migrating from high latitudes translocating nutrients to oligotrophic tropical systems (Roman et al., 2014). Thus, understanding the movement ecology and site selection of threatened species is not only crucial for informing effective management strategies, but also for gaining insights into their role in ecosystem function.

Many individuals repeatedly visited the same localized sites across many weeks, and we propose that manta rays likely locate these cleaning stations using conspicuous landmarks that they remember. Relatively little is known about effects of learning and memory on the movement patterns of marine megafauna (Hays et al., 2016). An unresolved question in marine megafauna movement ecology is how learning and memory or innate behaviors drive animal movements. It is likely that manta rays have a cognitive map of particular reef areas, akin to how animals with distinct home ranges know their environment intimately (Harten et al., 2020). In common with other taxa such as sea turtles, that alternate between oceanic and coastal areas, manta rays likely use coarse‐scale navigational cues in the open ocean, and precisely orientated movement in coastal areas (Hays et al., 2020). Manta rays visit the same reef systems across many years, as demonstrated by photo‐ID records (Couturier et al., 2014; Harris et al., 2020), and such repeated site use is similar to that seen in sea turtles and many bird species that maintain strong site fidelity to particular areas interspersed with long‐distance migration (Alerstam et al., 2006; Armstrong et al., 2019; Shimada et al., 2020). These observations imply that many migratory taxa, including manta rays, have a long‐term memory of particular focal sites.

We found that *M. alfredi* prefer hard coral structure, rather than soft coral cover or continuous coral ridge substrate, and that cleaner wrasse density was also more associated with this type of substrate. Cleaner species often use prominent coral heads or outcrops to set up their cleaning stations (Côté et al., 1998), and choosing a conspicuous location is likely to be beneficial to them for attracting clients. But for large, mobile species such as *M. alfredi*, these structures also provide suitable habitat to allow maneuverability and facilitate cleaning interactions. As for many other pelagic elasmobranch species, *M. alfredi* is a ram ventilator and has to swim continuously to irrigate its gills for uptake of oxygen. They are unable to rest on the bottom to facilitate cleaning interactions, as do some demersal elasmobranch species (Keyes, 1982; Sazima & Moura, 2000). Hard corals are particularly susceptible to the impacts of climate change, and the potential for coral reefs to recover from multiple stressors is declining (Hughes et al., 2018). Given the preference for hard coral structures, climate change could present a threat to the habitat of numerous cleaner species. Loss of habitat for cleaner species could have downstream consequences on the movements and site selection of large mobile clients like manta rays.

Wind conditions and tidal cycles are known to influence the movements of large animals, and this study also confirmed the importance of these environmental variables. There were contrasting patterns in how Wind Direction influenced reef manta ray Counts and the Duration of their visits, and this may be explained by the location of the study site on the western side of the island. Previous work has suggested manta rays favor this side of the island due to the shelter provided from prevailing winds (Couturier et al., 2018), and it may be that south to south‐westerly winds are more favorable for longer cleaning station attendance, but that the protection afforded on this side of the island means manta rays may be detected during other wind regimes as well. We found that Wind Speed influenced the Duration of visits here, but did not impact their detection rate, supporting that manta rays are still present during less favorable conditions but for shorter periods of time. Foraging opportunities may present a reason for the Tidal influence of manta ray detections at the site. Prior research has shown zooplankton concentrations in the vicinity of the current study site are found to peak prior to low tide, and manta rays are more commonly observed feeding during this tidal phase (Armstrong et al., 2016). It may be that manta rays attend the cleaning station for longer periods either side of this foraging opportunity. Tide and current movements have been implicated for cleaning behavior at other locations (Murie et al., 2020; O’Shea et al., 2010; Rohner et al., 2013), as moderate currents are favorable to a manta ray's ability to hold station and facilitate cleaning. This suggests that the preference of *M. alfredi* for prominent hard coral structures in the current study may be related both to suitable habitat for the cleaner wrasse and the hydrodynamics of the location that facilitates cleaning interactions.

We found a clear diurnal signal for *M. alfredi* attendance within the study region. First arrival occurred in the morning, after sunrise, and individual visits gradually declined throughout the day. Early in the day was also when individuals were more likely to spend longer periods of time in the cleaning station area. Similar findings have been reported for manta rays in other regions (Venables et al., 2020) and for other elasmobranchs (Oliver et al., 2011). Diurnal visitation likely reflects the behavior of the cleaner fish, since *L*. *dimidiatus* individuals are inactive at night, and do not return to their cleaning station habitats until after dawn (Potts, 1973). However, it is also likely a product of the behavior of the manta rays themselves, as they move offshore to forage at night. Satellite tracking has revealed night‐time diving behavior in manta rays (Braun et al., 2014; Stewart et al., 2016), and investigations using stable isotope analysis have suggested manta rays source their food from deep, benthic or epipelagic environments (Burgess et al., 2016; Couturier et al., 2013; Peel, Daly, et al., 2019). Planktivores from a range of taxa exhibit diurnal patterns in foraging behavior (Brierley, 2014; Hays, 2003), to take advantage of diel vertical migrating zooplankton that come into shallower waters at night.

We showed the utility of automated high‐accuracy acoustic tracking, which contrasts with historic active acoustic tracking that often has low accuracy, is limited in time and is labor intensive (Nelson et al., 1997). Levels of location accuracy we achieved in a reef environment (within a few meters) are similar to that recorded by others using the VPS approach in freshwater (Espinoza et al., 2011; Roy et al., 2014). By comparison, modern satellite tracking approaches such as Fastloc‐GPS, where locations are typically within a few 10s of meters of the true location (Dujon et al., 2014; Thomson et al., 2017), can provide continuous broad‐scale tracking at the cost of such precision. These modern approaches are transforming our understanding of the patterns of small‐scale space use for a range of marine species. For manta rays, a blended use of acoustic arrays and satellite tracking would provide a comprehensive understanding of space use over a range of spatial scales, and further clarify links between cleaning stations and adjacent feeding grounds (Jaine et al., 2014).

Combining tagging methods could also offer redundancy for tag failure or loss. Almost 20% of the tags that we deployed were never detected after their initial deployment, likely because of animal migration, tag shedding or tag failure. Understanding why transmitters stop relaying data is important to help drive improvements to tag design and deployment (Hays et al., 2007). Three of the eight tagged manta rays that were not detected by the VPS array were identified at the site by photo‐ID within the study period, showing that for these individuals at least, the issue was tag shedding or failure (tag attachment was not confirmed via photo‐ID). Nevertheless, for 34 individuals (81% of those tagged), tracking revealed repeated visits to cleaning areas. This large sample size, when compared to many tracking studies from a recent review (Sequeira et al., 2019), suggests that fidelity to cleaning areas is a general feature of manta ray ecology.

We hypothesize from the findings of the current study, together with other recent work (Murie et al., 2020; Stevens, 2016), that preferred cleaning station sites are likely paired with rich feeding grounds nearby. The current study location is about seven kilometers from the shelf edge, and the mesoscale oceanographic feature of the Capricorn Eddy (Weeks et al., 2010). The productivity of the Capricorn Eddy is a result of increased frontal activity and upwelling, providing foraging opportunities to seabirds such as wedge‐tailed shearwaters *Puffinus pacificus* (McDuie et al., 2018), and has been associated with foraging of manta rays (Jaine et al., 2014). Cleaning station environments, where manta rays sometimes stay in close proximity for long periods (i.e., weeks to months), would need to be in the vicinity of places that fulfill the multiple biological and ecological functions of these animals. Therefore, the feeding‐cleaning hypothesis—where mobile species select cleaning sites close to productive foraging opportunities—may also explain the habitat preferences of other large, mobile client species.

## CONFLICT OF INTEREST

The authors declare no conflict of interest.

## AUTHOR CONTRIBUTIONS

**Asia O. Armstrong:** Conceptualization (lead); Data curation (lead); Formal analysis (lead); Investigation (lead); Methodology (lead); Project administration (supporting); Resources (supporting); Supervision (supporting); Visualization (lead); Writing‐original draft (lead); Writing‐review & editing (lead). **Amelia J. Armstrong:** Conceptualization (supporting); Investigation (supporting); Methodology (supporting); Writing‐review & editing (supporting). **Michael B. Bennett:** Conceptualization (supporting); Funding acquisition (supporting); Methodology (supporting); Project administration (supporting); Supervision (supporting); Writing‐review & editing (supporting). **Anthony J. Richardson:** Data curation (supporting); Formal analysis (supporting); Funding acquisition (supporting); Investigation (supporting); Methodology (supporting); Supervision (supporting); Validation (supporting); Visualization (supporting); Writing‐review & editing (supporting). **Kathy A. Townsend:** Conceptualization (supporting); Funding acquisition (supporting); Investigation (supporting); Methodology (supporting); Resources (supporting); Supervision (supporting); Writing‐review & editing (supporting). **Jason D. Everett:** Data curation (supporting); Formal analysis (supporting); Visualization (supporting); Writing‐review & editing (supporting). **Graeme C. Hays:** Methodology (supporting); Visualization (supporting); Writing‐review & editing (supporting). **Hugh Pederson:** Conceptualization (supporting); Data curation (supporting); Methodology (supporting); Validation (supporting); Visualization (supporting); Writing‐review & editing (supporting). **Christine L. Dudgeon:** Conceptualization (supporting); Investigation (supporting); Methodology (supporting); Project administration (supporting); Resources (supporting); Supervision (supporting); Writing‐review & editing (supporting).

### OPEN RESEARCH BADGES

This article has earned an Open Data Badge for making publicly available the digitally‐shareable data necessary to reproduce the reported results. The data is available at https://doi.org/10.14264/2e79b25.

## Data Availability

Detection logs from the acoustic array have been archived on the UQ eSpace repository, accessible via the following link: https://doi.org/10.14264/2e79b25

## Appendix

**TABLE A1:** Metadata of manta ray tag deployments. Locations: NSI = North Stradbroke Island, LEI = Lady Elliot Island. Manta ID refers to the number assigned to each individual *M. alfredi* based on a long‐term photographic identification sighting database

Date	Location	Tag serial #	Tag ID	Manta ID	Sex	Maturity	Size estimate
4‐Feb‐17	NSI	1256553	51375	#0460	Female	Mature	3.5 m
4‐Feb‐17	NSI	1256554	51376	#0512	Female	Juvenile	3 m
04‐Feb‐17	NSI	1256555	51377	#0815	Female	Mature	3.5 m
04‐Feb‐17	NSI	1256556	51378	#1139	Male	Mature	2.5 m
04‐Feb‐17	NSI	1256557	51379	#0476	Female	Mature	3.5 m
04‐Feb‐17	NSI	1256558	51380	#0601	Female	Juvenile	3 m
05‐Feb‐17	NSI	1256559	51381	#0622	Female	Mature	3.5 m
05‐Feb‐17	NSI	1256560	51382	#0875	Male	Juvenile	3 m
05‐Feb‐17	NSI	1256561	51383	#0724	Female	Juvenile	3 m
05‐Feb‐17	NSI	1256562	51384	#0516	Female	Juvenile	3 m
28‐Feb‐17	LEI	1256563	51385	#0445	Female	Mature	3.5 m
28‐Feb‐17	LEI	1256564	51386	#1129	Female	Mature	3.5 m
03‐Jun‐17	LEI	1256565	51387	#0831	Male	Mature	3 m
04‐Jun‐17	LEI	1256566	51388	#0076	Female	Juvenile	3 m
06‐Jun‐17	LEI	1256567	51389	#1103	Female	Mature	3.5 m
06‐Jun‐17	LEI	1266921	32432	#0648	Female	Mature	3.5 m
06‐Jun‐17	LEI	1266922	32433	#0201	Female	Mature	3.5 m
06‐Jun‐17	LEI	1266923	32434	#0029	Female	Mature	3.5 m
06‐Jun‐17	LEI	1266924	32435	#0003	Female	Mature	3.5 m
07‐Jun‐17	LEI	1266925	32436	#0537	Male	Mature	3 m
07‐Jun‐17	LEI	1266926	32437	#0816	Female	Mature	3.5 m
07‐Jun‐17	LEI	1266927	32438	#0551	Female	Juvenile	3 m
07‐Jun‐17	LEI	1266928	32439	#0524	Female	Mature	3.5 m
07‐Jun‐17	LEI	1266929	32440	#0778	Male	Juvenile	<3 m
07‐Jun‐17	LEI	1266930	32441	#0654	Female	Juvenile	<3 m
08‐Jun‐17	LEI	1266931	32442	#0172	Male	Mature	<3 m
08‐Jun‐17	LEI	1266932	32443	#0313	Female	Juvenile	3 m
08‐Jun‐17	LEI	1266933	32444	#0454	Female	Mature	3.5 m
09‐Jun‐17	LEI	1266934	32445	#0021	Male	Mature	<3 m
09‐Jun‐17	LEI	1266935	32446	#0304	Male	Mature	3 m
09‐Jun‐17	LEI	1266936	32447	#0541	Female	Mature	4 m
09‐Jun‐17	LEI	1266937	32448	#0010	Female	Mature	3.5 m
10‐Jun‐17	LEI	1266938	32449	#0305	Female	Mature	3.5 m
10‐Jun‐17	LEI	1266939	32450	#0203	Male	Mature	3 m
11‐Jun‐17	LEI	1266940	32451	#0118	Male	Juvenile	<3 m
12‐Jun‐17	LEI	1266941	32452	#0034	Female	Mature	3.5 m
12‐Jun‐17	LEI	1266942	32453	#1133	Male	Mature	3 m
12‐Jun‐17	LEI	1266943	32454	#0297	Female	Mature	3.5 m
13‐Jun‐17	LEI	1266944	32455	#0228	Male	Mature	3 m
13‐Jun‐17	LEI	1266945	32456	#0725	Male	Mature	3 m
29‐Jun‐18	LEI	1278726	15737 (V16P)	#1260	Male	Mature	3.5m
17‐Jul‐18	LEI	1278727	15738 (V16P)	#0419	Male	Mature	3 m
